# Hepatitis A virus vaccination in Iranian soldiers needs more attention

**Published:** 2016

**Authors:** Seyed Moayed Alavian

**Affiliations:** 1 *Baqiyatallah Research Center for Gastroenterology and Liver Disease, Baqiyatallah University of Medical Sciences, Tehran, Iran*; 2 *Middle East Liver Disease Centre, Tehran, Iran*

**To The Editor:**

I read with interest the recent published article (1) in your esteemed journal. The Presented data regarding the prevalence of hepatitis A virus (HAV) infection in this paper was very interesting. However, I would like to highlight some points for more clarification of the issue.

The prevalence of HAV infection differs in various parts of the world based on the geographic area, sanitary conditions and socioeconomic levels (2). There are several reports about shifting epidemiological pattern of HAV infection from high prevalence to lower endemicity as a result of improved living conditions from all over the world, even in underdeveloped and developing countries, as well as Iran (3, 4). There are also many conflicts such as war, floods and crisis in our region affecting access to safe water and proper disposal of wastes (5). Additionally, travelling to holy places such as Iraq and Syria, can increase the risk of infection. Recently, Ghasemian, et al. in their paper, reported nine patients with acute HAV infection after returning from Karbala to Iran (6). Therefore, it sensitizes us to be more careful, especially regarding soldiers who are going to Iraq and Syria for combatting the terrorism. Contaminated water may be a potential source for the spread of this transmissible enteric disease. Therefore, it is mandatory to educate soldiers about prevention methods and consider vaccination against HAV. 
